# Safety of two-dose COVID-19 vaccination (BNT162b2 and CoronaVac) in adults with cancer: a territory-wide cohort study

**DOI:** 10.1186/s13045-022-01265-9

**Published:** 2022-05-19

**Authors:** Wei Kang, Jessica J. P. Shami, Vincent K. C. Yan, Xuxiao Ye, Joseph E. Blais, Xue Li, Victor H. F. Lee, Celine S. L. Chui, Francisco T. T. Lai, Eric Y. F. Wan, Carlos K. H. Wong, Ian C. K. Wong, Esther W. Chan

**Affiliations:** 1 Centre for Safe Medication Practice and Research, Department of Pharmacology and Pharmacy, General Office, L02-56 2/F, Laboratory Block, LKS Faculty of Medicine, The University of Hong Kong, 21 Sassoon Road, Pokfulam, Hong Kong SAR, China; 2 Laboratory of Data Discovery for Health, Hong Kong SAR, China; 3 Department of Medicine, LKS Faculty of Medicine, The University of Hong Kong, Hong Kong SAR, China; 4 Department of Clinical Oncology, LKS Faculty of Medicine, Queen Mary Hospital, The University of Hong Kong, Hong Kong SAR, China; 5 School of Nursing, LKS Faculty of Medicine, The University of Hong Kong, Hong Kong SAR, China; 6 School of Public Health, LKS Faculty of Medicine, The University of Hong Kong, Hong Kong SAR, China; 7 Department of Family Medicine and Primary Care, LKS Faculty of Medicine, The University of Hong Kong, Hong Kong SAR, China; 8 Research Department of Practice and Policy, School of Pharmacy, University College London, London, UK; 9 Department of Pharmacy, The University of Hong Kong-Shenzhen Hospital, Shenzhen, China; 10 The University of Hong Kong Shenzhen Institute of Research and Innovation, Shenzhen, China

**Keywords:** COVID-19, Vaccine, Safety, Adverse events of special interest (AESI), BNT162b2, CoronaVac, Cancer

## Abstract

**Background:**

The World Health Organization has defined a list of adverse events of special interest (AESI) for safety surveillance of vaccines. AESI have not been adequately assessed following COVID-19 vaccination in patients with cancer contributing to vaccine hesitancy in this population. We aimed to evaluate the association between BNT162b2 and CoronaVac vaccines and the risk of AESI in adults with active cancer or a history of cancer.

**Patients and methods:**

We conducted a territory-wide cohort study using electronic health records managed by the Hong Kong Hospital Authority and vaccination records provided by the Department of Health. Patients with a cancer diagnosis between January 1, 2018, and September 30, 2021, were included and stratified into two cohorts: active cancer and history of cancer. Within each cohort, patients who received two doses of BNT162b2 or CoronaVac were 1:1 matched to unvaccinated patients using the propensity score. Cox proportional hazards regression was used to estimate hazard ratios (HR) and 95% confidence intervals (CIs) for AESI 28 days after the second vaccine dose.

**Results:**

A total of 74,878 patients with cancer were included (vaccinated: 25,789 [34%]; unvaccinated: 49,089 [66%]). Among patients with active cancer, the incidence of AESI was 0.31 and 1.02 per 10,000 person-days with BNT162b2 versus unvaccinated patients and 0.13 and 0.88 per 10,000 person-days with CoronaVac versus unvaccinated patients. Among patients with history of cancer, the incidence was 0.55 and 0.89 per 10,000 person-days with BNT162b2 versus unvaccinated patients and 0.42 and 0.93 per 10,000 person-days with CoronaVac versus unvaccinated patients. Neither vaccine was associated with a higher risk of AESI for patients with active cancer (BNT162b2: HR 0.30, 95% CI 0.08–1.09; CoronaVac: 0.14, 95% CI 0.02–1.18) or patients with history of cancer (BNT162b2: 0.62, 95% CI 0.30–1.28; CoronaVac: 0.45, 95% CI 0.21–1.00).

**Conclusions:**

In this territory-wide cohort study of patients with cancer, the incidence of AESI following vaccination with two doses of either BNT162b2 or CoronaVac vaccines was low. The findings of this study can reassure clinicians and patients with cancer about the overall safety of BNT162b2 and CoronaVac in patients with cancer, which could increase the COVID-19 vaccination rate in this vulnerable group of patients.

**Supplementary Information:**

The online version contains supplementary material available at 10.1186/s13045-022-01265-9.

## Introduction

Public health agencies recommend that patients with cancer should be prioritized for COVID-19 vaccination [1–3]. Currently, the safety of COVID-19 vaccines remains a concern, especially among the elderly and immunocompromised patients such as patients with cancer [4]. This has led to lower rates of vaccine uptake in patients with cancer in some regions including Hong Kong [5–7]. However, the available observational studies of BNT162b2 (mRNA, Pfizer-BioNTech) and CoronaVac (inactivated, Sinovac) vaccines in patients with cancer have only assessed common adverse events, for example headache and fever; have small sample sizes and are therefore unable to detect uncommon or rare adverse events of special interest (AESI); and do not have suitable between-individual comparisons, since they either have no comparator group or use a comparator group of healthy adults without cancer [8–15]. Furthermore, most patients with cancer were excluded from pivotal clinical trials of BNT162b2 and CoronaVac as their cancer treatments may suppress or impair the immune system [16–18]. Our study aimed to describe and assess the risk of AESI, as defined by the World Health Organization, among patients with active cancer and a history of cancer who received vaccination with BNT162b2 or CoronaVac.

## Methods

### Data sources

This study used electronic health records provided by the Hospital Authority and linked vaccination records provided by the Department of Health in Hong Kong. The linked records have been previously used to evaluate the safety of COVID-19 vaccines [19–23]. Diagnosis records were identified using the International Classification of Diseases, Ninth Revision, Clinical Modification (ICD-9-CM) diagnosis codes (Additional file 1: Table S1), and prescription records were identified using British National Formulary (BNF) codes (Additional file 1: Table S2).

### Study population

**Fig. 1:**
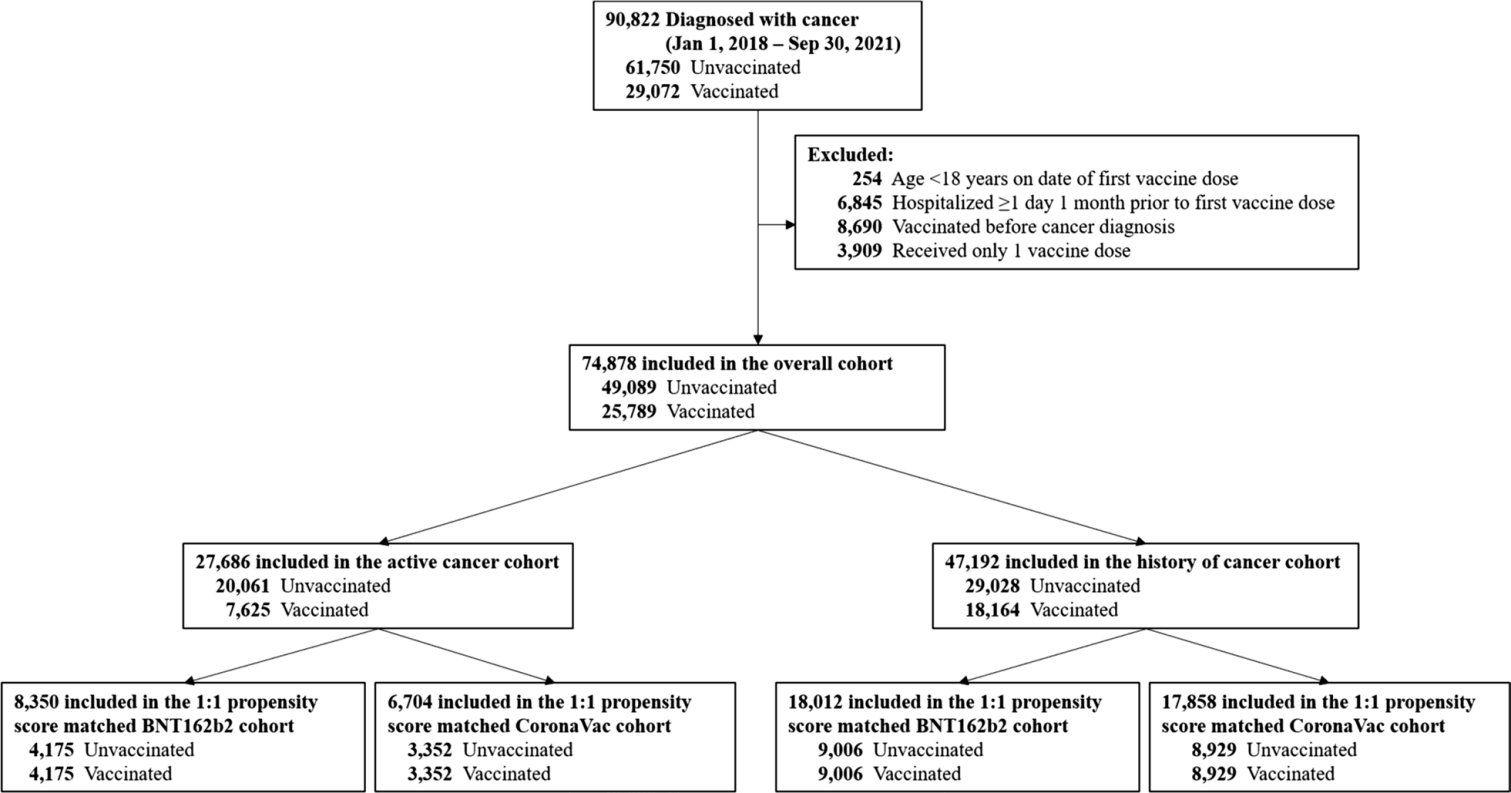
Patient selection flowchart

Patients with a cancer diagnosis record between January 1, 2018, and September 30, 2021, were identified. Since patients with cancer have a weaker immune response after COVID-19 vaccination, AESI outcomes were only evaluated following the second dose of the vaccine [24]. The index date was defined as the date of the second vaccine dose for patients who were vaccinated with either BNT162b2 or CoronaVac. For unvaccinated patients, the pseudo index date was selected from a corresponding vaccine recipient matched on age and sex. Patients younger than 18 years, hospitalized within 30 days before vaccination, or diagnosed with cancer on or after the first dose of vaccination were excluded. Patients who received only the first dose of the vaccine were also excluded. The study population was stratified into two mutually exclusive cohorts: patients with active cancer and patients with a history of cancer (Fig. 1). Active cancer patients were defined as those who had undergone any active cancer treatment or had a diagnosis of metastasis in the last 6 months before their first vaccine dose [25]. The remaining patients were considered as the history of cancer cohort.

### Outcomes

The primary outcome of interest in this study was the incidence of 28-day AESI, defined by the World Health Organization as a list of important vaccine safety surveillance events. The list includes conditions such as acute respiratory distress syndrome, acute kidney injury, myocarditis, and thrombocytopenia (Additional file 1: Table S1) [26]. The secondary outcome was 28-day all-cause mortality. Patients were followed from the index date until a diagnosis of the outcome, death, 28 days after the index date, or the end of study period (September 30, 2021), whichever occurred first.

### Statistical analysis

Baseline patient characteristics were presented as means (standard deviation) for continuous variables and frequencies (percentages) for categorical variables. To reduce confounding arising from differences in baseline characteristics between vaccinated and unvaccinated patients, propensity score (PS) matching was performed for each type of vaccine (both in active cancer and in history of cancer cohorts). Confounders included in the PS estimation included age, sex, smoking, obesity, index date, history of COVID-19 (history of positive PCR test), latest levels of white blood cells and neutrophils before vaccination, hospitalization, accident and emergency attendance, cancer type and site, comorbidities, and concomitant medication use (Additional file 1: Tables S3, S4). Patients who received BNT162b2 vaccine and unvaccinated patients were matched on a 1:1 ratio using nearest neighbor algorithm with a caliper of 0.01. The same matching procedure was performed for patients who received the CoronaVac vaccine. A standardized mean difference (SMD) of < 0.1 was considered acceptable.

The association of AESI with either BNT162b2 or CoronaVac vaccine among patients with cancer was estimated using Cox proportional hazards regression. The results were reported as hazard ratios (HR) with 95% confidence intervals (CIs). Subgroup analyses were performed on different age-groups, sex, and cancer types. Individuals who experienced severe adverse effects after the first dose would less likely accept the second dose, which could potentially introduce bias in the current two-dose analysis. Hence, a post hoc analysis was conducted to compare the cumulative incidence rate of AESI between patients who received one dose only and unvaccinated patients; chi-square test with a significance level of 0.05 was reported.

R version 4.0.3 (R Foundation for Statistical Computing, Vienna, Austria) was used for all statistical analyses. The analyses were conducted by WK and cross-checked independently by JJPS and XY for quality assurance.

## Results

We identified 90,822 patients with a cancer diagnosis between January 1, 2018, and September 30, 2021. After applying the exclusion criteria, 74,878 patients (25,789 active cancer cohort and 49,089 history of cancer cohort) were included (Fig. 1). After 1:1 PS matching, 15,054 patients with active cancer (4175 BNT162b2; 3352 CoronaVac; 7527 unvaccinated) and 35,870 patients with a history of cancer (9006 BNT162b2; 8929 CoronaVac; 17,935 unvaccinated) were included (Additional file 1: Tables S3, S4). All SMDs of the variables were < 0.1.

In the active cancer cohort, the incidence of AESI was 0.31 and 1.02 per 10,000 person-days for patients receiving BNT162b2 and matched unvaccinated patients, respectively; 0.13 and 0.88 per 10,000 person-days for patients receiving CoronaVac vaccine and matched unvaccinated patients, respectively (Table 1, Additional file 1: Table S5). In patients with a history of cancer, the incidence of AESI was 0.55 and 0.89 per 10,000 person-days for those who received BNT162b2 and matched unvaccinated patients, respectively; 0.42 and 0.93 per 10,000 person-days for patients who received CoronaVac and matched unvaccinated patients, respectively.

**Table 1:** Risk of 28-day post-vaccination AESI in vaccinated and unvaccinated patients with cancer after propensity score matching

Cohorts	BNT162b2				CoronaVac			
	Events/follow-up time (person-days)/incidence (per 10,000 person-days)		Hazard ratio^a^ (95% CI)	*P* value	Events/follow-up time (person-days)/incidence (per 10,000 person-days)		Hazard ratio (95% CI)	*P* value
	Unvaccinated (*N* = 4175)	Vaccinated (*N* = 4175)			Unvaccinated (*N* = 3352)	Vaccinated (*N* = 3352)		
*Active cancer*								
All	10/97586/1.02	3/97588/0.31	0.30 (0.08–1.09)	0.07	7/79150/0.88	1/78204/0.13	0.14 (0.02–1.18)	0.07
Male	7/24853/2.82	1/23796/0.42	0.15 (0.02–1.22)	0.08	3/23137/1.30	1/22894/0.44	–	–
Female	3/72733/0.41	2/73792/0.27	0.65 (0.11–3.92)	0.64	4/56013/0.71	0/55310/0	–	–
Age < 60 years	1/50970/0.20	2/51891/0.39	–	–	1/33853/0.30	1/33410/0.30	–	–
Age ≥ 60 year	9/46616/1.93	1/45697/0.22	0.11 (0.01–0.90)	< 0.05	6/45297/1.32	0/44794/0	–	–
Solid tumor	8/90350/0.89	3/90634/0.33	0.37 (0.10–1.41)	0.15	7/75622/0.93	1/74229/0.13	0.15 (0.02–1.19)	0.07
Hematological malignancy	2/7236/2.76	0/6954/0	–	–	0/3528/0	0/3975/0	–	–

	Unvaccinated (*N* = 9006)	Vaccinated (*N* = 9006)			Unvaccinated (*N* = 8929)	Vaccinated (*N* = 8929)		
*History of cancer*								
All	19/213182/0.89	12/216640/0.55	0.62 (0.30–1.28)	0.20	20/214652/0.93	9/213033/0.42	0.45 (0.21–1.00)	< 0.05
Male	13/94638/1.37	4/96765/0.41	0.30 (0.10–0.92)	< 0.05	14/107447/1.30	8/106183/0.75	0.58 (0.24–1.38)	0.22
Female	6/118544/0.51	8/119875/0.67	1.32 (0.46–3.81)	0.61	6/107205/0.56	1/106850/0.09	0.17 (0.02–1.39)	0.10
Age < 60 years	7/99158/0.71	4/99283/0.40	0.57 (0.17–1.96)	0.37	5/77057/0.65	0/74134/0	–	–
Age ≥ 60 years	12/114024/1.05	8/117357/0.68	0.65 (0.27–1.60)	0.35	15/137595/1.09	9/138899/0.65	0.59 (0.26–1.36)	0.22
Solid tumor	18/197988/0.91	10/202083/0.49	0.55 (0.25–1.18)	0.12	19/203168/0.94	8/201881/0.40	0.42 (0.19–0.97)	< 0.05
Hematological malignancy	1/15194/0.66	2/14557/1.37	–	–	1/11484/0.87	1/11152/0.90	–	–

*AESI:* adverse events of special interest

^a^Hazard ratios are not shown if total events are less than 5 in each subgroup

Patients who received BNT162b2 or CoronaVac were not at a higher risk of AESI compared to unvaccinated patients in the active cancer cohort [BNT162b2 HR: 0.30 (95% CI 0.08–1.09); CoronaVac HR: 0.14 (95% CI 0.02–1.18)]. Similarly, patients who received BNT162b2 or CoronaVac were not at a higher risk of AESI compared to unvaccinated patients in the history of cancer cohort [BNT162b2 HR: 0.62 (95% CI 0.30–1.28), CoronaVac HR: 0.45 (95% CI 0.21–1.00)] (Table 1). Results were consistent in all subgroup analyses; vaccinated patients had no increased risk of AESI compared to unvaccinated patients.

Among patients with active cancer, there were two deaths in the BNT162b2 group versus 22 among matched unvaccinated patients; and no deaths in the CoronaVac group versus 12 among matched unvaccinated patients. Among patients with a history of cancer, there was one death in the BNT162b2 group versus 13 among matched unvaccinated patients, and 2 deaths in the CoronaVac group versus 17 among matched unvaccinated patients (Additional file 1: Table S5). In the post hoc analysis, the cumulative incidence rate of AESI was not significantly different between patients who received one dose only, compared to unvaccinated patients (0.5% one-dose only and 0.4% unvaccinated, χ^2^ = 0.63, *p* = 0.43; Additional file 1: Table S6).

## Discussion

The low rate of COVID-19 vaccine uptake in our study appears to reflect safety concerns among patients with cancer in Hong Kong. On September 30, 2021, our data showed that the overall vaccination rate in Hong Kong was 58.8%, while among patients with cancer it was only 30.2%. Our study provides reassurance that patients with cancer are not at an increased risk of AESI or death following two doses of either BNT162b2 or CoronaVac.

Several small observational studies have evaluated the safety of BNT162b2 or CoronaVac vaccines in patients with cancer [8–15]. All of those studies evaluated short-term common adverse events, including pain and swelling at the injection site, headache, fever, and diarrhea. However, no previous study examined AESI as an outcome and none included both patients with active cancer and patients with a history of cancer. To date, the largest study included 816 patients with active cancer and 274 healthcare workers from a single institution in Italy [9]. However, the comparator group comprised healthy individuals with no cancer diagnosis.

To our knowledge, this is the first study to report on all AESI and to evaluate the association between BNT162b2 and CoronaVac and the risk of AESI among patients with active cancer or history of cancer. Our study is also the first and largest territory-wide cohort study that reports on 25,789 patients vaccinated with either BNT162b2 or CoronaVac. Furthermore, our study provides reassuring safety data on these two vaccines in a predominantly Asian population.

This study has several limitations. Firstly, patients in relatively better health or with better prognosis are more likely to get vaccinated, which may lead to a healthy user bias. Therefore, PS matching was used to minimize baseline confounding. Secondly, most AESI that were examined tend to be severe and relatively rare (< 1/1000 person-years) [27]. As a result, we would have been unable to detect a small increase in AESI risk. Nevertheless, the findings are still reassuring since the number of AESI events was small. Finally, since patients who only received the first dose of the vaccine were excluded, this could bias the current two-dose analysis. Nevertheless, our post hoc analysis did not show any significant difference in the cumulative incidence rate of AESI between patients receiving one dose only compared to unvaccinated patients; hence, this is unlikely to bias our findings [28, 29]. Future studies with a longer follow-up period are needed to further inform about potential longer-term risks.

## Conclusion

In Hong Kong, the vaccination rate among patients with cancer is relatively low. In the present study, there was no increased risk of AESI following two doses of either BNT162b2 or CoronaVac vaccines among patients with active cancer or a history of cancer. The findings of this study can reassure clinicians and patients about the overall short-term safety of BNT162b2 and CoronaVac in patients with cancer, which could increase the COVID-19 vaccination rate in this vulnerable group of patients.

## Supplementary Information

**Additional file 1.** Supplementary figures.

## Data Availability

The datasets analyzed during the current study are not publicly available.

## Abbreviations

AESI: Adverse events of special interest
ICD-9-CM: International Classification of Diseases, Ninth Revision, Clinical Modification
BNF: British National Formulary
PS: Propensity score
HR: Hazard ratio
CI: Confidence interval
SMD: Standardized mean difference
