# Supplementary material for: Safety of two-dose COVID-19 vaccination (BNT162b2 and CoronaVac) in adults with cancer: a territory-wide cohort study
Source: J Hematol Oncol. 2022 May 19;15:66. doi: 10.1186/s13045-022-01265-9 (PMC9117990; doi:10.1186/s13045-022-01265-9)
Supplement: Supplementary file 1 — Additional file 1. Supplementary figures. [file 13045_2022_1265_MOESM1_ESM.docx]

**Table S1. List of diagnosis codes used in the study**

| **Comorbidities** | **ICD-9-CM diagnosis codes** |
| --- | --- |
| Cancer | 140-209, 230-234 |
| Carcinoma | 230-234 |
| Metastasis | 196-198 |
| Cancer site |  |
| Bladder | 188 |
| Breast | 174-175 |
| Colorectum | 153-154 |
| Liver | 155 |
| Lung | 162 |
| Nasopharynx | 147 |
| Prostrate | 185 |
| Skin | 172-173 |
| Stomach | 151 |
| Thyroid | 193 |
| Uterus | 179-180, 182 |
| Hematological malignancies | 200-208 |
| Solid tumor | 140-199, 209, 230-234 |
| Smoking | V15.82, 305.1 |
| Obesity | 278.0, V85.3-4 |
| Congestive Heart Failure | 398.91, 402.01, 402.0.11, 402.91, 404.01, 404.03, 404.11, 404.13, 404.91, 404.93, 428 |
| Hypertension | 401-405, 437.2 |
| Diabetes | 250 |
| Systemic embolism | 444-445 |
| Vascular disease | 410-414, 443.8-443.9 |
| Peripheral vascular disease | 441, 443.9, 785.4, V43.4 |
| Cerebrovascular disease | 430-438 |
| Chronic obstructive pulmonary disease | 490-496, 500-505, 506.4 |
| Dementia | 290 |
| Paralysis | 342, 344.1 |
| Chronic renal failure | 582, 585, 586, 588, 583.0-583.2, 583.4, 583.6, 583.7 |
| Liver diseases | 456.0-456.2, 571.2, 571.24-571.26, 572.2-572.4, 572.8 |
| Ulcers | 531-534 |
| Rheumatoid arthritis and other inflammatory polyarthropathies | 710.0, 710.1, 710.4, 714.0-714.2, 714.81, 725 |
| Respiratory infections | 460-466, 480-488 |
| Viral infections | 053, 054, 058, 088.81, 042, 483.0 |
| Migraine | 346 |
| Mental health disorders | 290-319 |
| AESI |  |
| Guillain-Barré Syndrome | 357.0, 357.8, 357.9, N94 |
| Acute disseminated encephalomyelitis (ADEM) | 323.6, 323.8 |
| Narcolepsy | 347, 89.17, 89.18, 307.4, 780.5 |
| Acute aseptic arthritis | 274.0, 696.0, 716.5, 716.6, 716.9, 712, 711.5 |
| Type 1 Diabetes | 250.01-250.03, 250.11, 250.13, 250.21, 250.23, 250.31, 250.33, 250.41, 250.43, 250.51, 250.53, 250.61, 250.63, 250.71, 250.73, 250.81, 250.83 |
| (Idiopathic) Thrombocytopenia | 287.3-287.5, 279.12, 283.11, 284.1, 446.6, 776.1 |
| Subacute thyroiditis | 245.1 |
| Microangiopathy | 446.6 |
| Heart failure | 428, 398.91, 402.01, 402.11, 402.91, 404.01, 404.03, 404.11, 404.13, 404.91, 404.93, K77 |
| Stress cardiomyopathy | 429.83 |
| Coronary artery disease | 410-414, V45.81, 36.0, 36.1, K74-K76 |
| Arrhythmia | 427, 426, 794.3, 785.0, K79, K80 |
| Myocarditis | 422, 429.0, 420.9, 423.9, K84 |
| Thromboembolism | 415.1, 453, 443-445, 433-436, 437.0, 437.1, 437.6, 437.8, 437.9, 451, 452, 325, 286.6, 459.9, 557.0, 557.9, K89, K90, K91, K93, K94 |
| Hemorrhagic disease | 286.5, 286.7, 287, 430, 431, 432.0, 432.9, 99.06, K90, K91 |
| Single Organ Cutaneous Vasculitis | 709.1, 446.2, 287.0, B83, K99 |
| Acute liver injury | 570, 573.3, D80, D97 |
| Acute kidney injury | 584, 586, U99 |
| Acute pancreatitis | 577.0 |
| Generalized convulsion | 345, 780.3, 779.0, N07, N88 |
| Meningoencephalitis | 322.9, 323.0, 323.4-323.9, 330.8, 377.73, 046.3, 049.0, 049.8, 049.9, 036.1, 056.01, 136.2, 130, 054.3, 094.1, 072.2, 013.0, 062.4, 045.0, 062, N70, N71 |
| Transverse myelitis | 323.0, 323.4-323.6, 323.8, 341.2 |
| Bell's Palsy | 351.0, 351.8, 351.9, N91 |
| Acute respiratory distress syndrome | 518.5, 518.8, 96.7, R99 |
| Erythema multiforme | 695.1 |
| Chilblain - like lesions | 991.5, A88 |
| Anosmia, ageusia | 781.1, N16 |
| Anaphylaxis | 995.0, 995.1, 995.3, 995.4, 999.4, 708.9, 519.11, 786.1, 458.9, A12, A99, A84, A85 |
| Sudden death | 798-799 |
| Rhabdomyolysis | 728.88-728.89, 791.3 |

AESI: adverse events of special interest; ICD-9-CM: International Classification of Diseases, Ninth revision, Clinical Modification

**Table S2. List of drug codes used in the study**

| **Prescriptions** | **BNF codes** |
| --- | --- |
| Cancer treatment |  |
| [Cytotoxic drugs](https://openprescribing.net/bnf/0801/) | 8.1 |
| Immunosuppressants | 8.2 |
| [Sex hormones and hormone antagonists in malignant disease](https://openprescribing.net/bnf/0803/) | 8.3 |
| Renin-angiotensin-system agents | 2.5.5 |
| Beta blockers | 2.4 |
| Calcium channel blockers | 2.6.2 |
| Diuretics | 2.2 |
| Nitrates | 2.6.1 |
| Lipid lowering agents | 2.12 |
| Insulins | 6.1.1 |
| Antidiabetic drugs | 6.1.2 |
| Antiarrthymic drugs | 2.3.2 |
| Cardiac glycosides | 2.1.1 |
| Anticoagulants | 2.8 |
| Antiplatelets | 2.9 |
| Antifibrinolytics and hemostatic | 2.11 |
| Hormonal agents | 7.3, 6.4, 8.3 |
| Glucocorticoids | 6.3.2 |
| Antidepressants | 4.3 |
| Intravenous immunoglobulin | 14.5.1 |
| NSAIDs | 10.1.1 |
| Drugs for gout | 10.1.4 |
| Antiepileptic drugs | 4.8.1 |
| Antiviral drugs | 5.3 |
| Antibacterial drugs | 5.1 |
| Immunosuppressants | 8.2 |
| Adrenaline | 2.7.3 |

BNF: British National Formulary; NSAIDs: Non-steroidal anti-inflammatory drugs

**Table S3.** **Baseline characteristics of patients with active cancer before and after propensity score matching**

|  | Before matching | | | | Matched BNT162b2 | | | Matched CoronaVac | | |
| --- | --- | --- | --- | --- | --- | --- | --- | --- | --- | --- |
|  | Unvaccinated | BNT162b2 | CoronaVac | SMD | Unvaccinated | BNT162b2 | SMD | Unvaccinated | CoronaVac | SMD |
| n | 20061 | 4260 | 3365 |  | 4175 | 4175 |  | 3352 | 3352 |  |
| Age (mean (SD)), year | 65.40 (12.54) | 58.56 (11.68) | 61.67 (11.20) | 0.383 | 58.92 (12.94) | 58.95 (11.41) | 0.003 | 61.71 (12.93) | 61.74 (11.16) | 0.002 |
| Sex = M | 7439 (37.1) | 1040 (24.4) | 992 (29.5) | 0.184 | 1071 (25.7) | 1032 (24.7) | 0.022 | 992 (29.6) | 991 (29.6) | 0.001 |
| Smoking | 250 (1.2) | 17 (0.4) | 28 (0.8) | 0.063 | 13 (0.3) | 17 (0.4) | 0.016 | 34 (1.0) | 28 (0.8) | 0.019 |
| Obesity | 1112 (5.5) | 136 (3.2) | 157 (4.7) | 0.077 | 148 (3.5) | 136 (3.3) | 0.016 | 147 (4.4) | 157 (4.7) | 0.014 |
| Covid-19 | 32 (0.2) | 0 (0.0) | 0 (0.0) | 0.038 | 0 (0.0) | 0 (0.0) | <0.001 | 0 (0.0) | 0 (0.0) | <0.001 |
| White blood cells (mean (SD)), 10^9^/L | 6.08 (4.12) | 5.91 (2.15) | 6.05 (2.27) | 0.042 | 5.92 (2.51) | 5.91 (2.15) | 0.004 | 6.06 (2.44) | 6.05 (2.27) | 0.001 |
| Neutrophils (mean (SD)), x10^9^/L | 3.80 (2.31) | 3.64 (1.87) | 3.69 (1.85) | 0.054 | 3.66 (2.08) | 3.64 (1.88) | 0.013 | 3.77 (2.10) | 3.70 (1.86) | 0.039 |
| Counts of hospitalization since 1 Jan, 2018 (mean (SD)) | 8.98 (9.88) | 6.65 (8.64) | 5.92 (7.47) | 0.23 | 6.86 (7.80) | 6.70 (8.68) | 0.02 | 5.89 (6.58) | 5.94 (7.48) | 0.007 |
| Counts of Accident & Emergency attendance since 1 Jan, 2018 (mean (SD)) | 1.98 (2.49) | 1.08 (1.85) | 1.33 (2.10) | 0.271 | 1.11 (1.53) | 1.10 (1.87) | 0.008 | 1.34 (1.92) | 1.33 (2.11) | 0.008 |
| Carcinoma in situ | 94 (0.5) | 21 (0.5) | 24 (0.7) | 0.021 | 22 (0.5) | 21 (0.5) | 0.003 | 20 (0.6) | 23 (0.7) | 0.011 |
| Metastasis | 920 (4.6) | 71 (1.7) | 69 (2.1) | 0.113 | 64 (1.5) | 71 (1.7) | 0.013 | 74 (2.2) | 69 (2.1) | 0.01 |
| Cancer site |  |  |  |  |  |  |  |  |  |  |
| Bladder | 292 (1.5) | 47 (1.1) | 70 (2.1) | 0.052 | 54 (1.3) | 47 (1.1) | 0.015 | 69 (2.1) | 70 (2.1) | 0.002 |
| Breast | 7636 (38.1) | 2687 (63.1) | 2001 (59.5) | 0.343 | 2591 (62.1) | 2613 (62.6) | 0.011 | 1988 (59.3) | 1990 (59.4) | 0.001 |
| Colorectum | 2025 (10.1) | 171 (4.0) | 130 (3.9) | 0.164 | 158 (3.8) | 171 (4.1) | 0.016 | 121 (3.6) | 130 (3.9) | 0.014 |
| Liver | 495 (2.5) | 52 (1.2) | 53 (1.6) | 0.062 | 39 (0.9) | 52 (1.2) | 0.03 | 46 (1.4) | 53 (1.6) | 0.017 |
| Lung | 2550 (12.7) | 279 (6.5) | 224 (6.7) | 0.14 | 267 (6.4) | 278 (6.7) | 0.011 | 228 (6.8) | 224 (6.7) | 0.005 |
| Nasopharynx | 379 (1.9) | 34 (0.8) | 21 (0.6) | 0.076 | 28 (0.7) | 34 (0.8) | 0.017 | 20 (0.6) | 21 (0.6) | 0.004 |
| Prostrate | 1857 (9.3) | 325 (7.6) | 394 (11.7) | 0.092 | 353 (8.5) | 325 (7.8) | 0.025 | 397 (11.8) | 393 (11.7) | 0.004 |
| Skin | 89 (0.4) | 4 (0.1) | 8 (0.2) | 0.046 | 3 (0.1) | 4 (0.1) | 0.008 | 9 (0.3) | 8 (0.2) | 0.006 |
| Stomach | 320 (1.6) | 40 (0.9) | 23 (0.7) | 0.058 | 46 (1.1) | 40 (1.0) | 0.014 | 28 (0.8) | 23 (0.7) | 0.017 |
| Thyroid | 99 (0.5) | 23 (0.5) | 17 (0.5) | 0.004 | 19 (0.5) | 23 (0.6) | 0.014 | 19 (0.6) | 17 (0.5) | 0.008 |
| Uterus | 319 (1.6) | 33 (0.8) | 31 (0.9) | 0.051 | 31 (0.7) | 33 (0.8) | 0.005 | 41 (1.2) | 31 (0.9) | 0.029 |
| Solid tumor | 18424 (91.8) | 3952 (92.8) | 3191 (94.8) | 0.08 | 3864 (92.6) | 3877 (92.9) | 0.012 | 3197 (95.4) | 3178 (94.8) | 0.026 |
| Hematological malignancies | 1637 (8.2) | 308 (7.2) | 174 (5.2) | 0.08 | 311 (7.4) | 298 (7.1) | 0.012 | 155 (4.6) | 174 (5.2) | 0.026 |
| Comorbidities |  |  |  |  |  |  |  |  |  |  |
| Congestive Heart Failure | 274 (1.4) | 17 (0.4) | 18 (0.5) | 0.07 | 22 (0.5) | 17 (0.4) | 0.018 | 19 (0.6) | 18 (0.5) | 0.004 |
| Hypertension | 6686 (33.3) | 864 (20.3) | 867 (25.8) | 0.198 | 851 (20.4) | 864 (20.7) | 0.008 | 862 (25.7) | 867 (25.9) | 0.003 |
| Diabetes | 3476 (17.3) | 412 (9.7) | 444 (13.2) | 0.15 | 426 (10.2) | 412 (9.9) | 0.011 | 448 (13.4) | 444 (13.2) | 0.004 |
| Systemic embolism | 8 (0.0) | 1 (0.0) | 1 (0.0) | 0.006 | 1 (0.0) | 1 (0.0) | <0.001 | 0 (0.0) | 1 (0.0) | 0.024 |
| Vascular disease | 898 (4.5) | 102 (2.4) | 87 (2.6) | 0.076 | 110 (2.6) | 102 (2.4) | 0.012 | 77 (2.3) | 87 (2.6) | 0.019 |
| Peripheral vascular disease | 81 (0.4) | 9 (0.2) | 9 (0.3) | 0.023 | 9 (0.2) | 9 (0.2) | <0.001 | 9 (0.3) | 9 (0.3) | <0.001 |
| Cerebrovascular disease | 931 (4.6) | 61 (1.4) | 71 (2.1) | 0.127 | 44 (1.1) | 61 (1.5) | 0.037 | 78 (2.3) | 71 (2.1) | 0.014 |
| Chronic obstructive pulmonary disease | 587 (2.9) | 60 (1.4) | 60 (1.8) | 0.07 | 58 (1.4) | 60 (1.4) | 0.004 | 52 (1.6) | 60 (1.8) | 0.019 |
| Dementia | 66 (0.3) | 1 (0.0) | 3 (0.1) | 0.051 | 3 (0.1) | 1 (0.0) | 0.022 | 1 (0.0) | 3 (0.1) | 0.024 |
| Paralysis | 38 (0.2) | 1 (0.0) | 0 (0.0) | 0.045 | 0 (0.0) | 1 (0.0) | 0.022 | 0 (0.0) | 0 (0.0) | <0.001 |
| Chronic renal failure | 395 (2.0) | 33 (0.8) | 24 (0.7) | 0.073 | 27 (0.6) | 33 (0.8) | 0.017 | 20 (0.6) | 24 (0.7) | 0.015 |
| Ulcers | 479 (2.4) | 41 (1.0) | 41 (1.2) | 0.075 | 41 (1.0) | 41 (1.0) | <0.001 | 37 (1.1) | 41 (1.2) | 0.011 |
| Rheumatoid arthritis and other inflammatory polyarthropathies | 113 (0.6) | 20 (0.5) | 12 (0.4) | 0.02 | 19 (0.5) | 20 (0.5) | 0.004 | 15 (0.4) | 12 (0.4) | 0.014 |
| Respiratory infections | 1470 (7.3) | 166 (3.9) | 129 (3.8) | 0.102 | 155 (3.7) | 163 (3.9) | 0.01 | 123 (3.7) | 129 (3.8) | 0.009 |
| Viral infections | 207 (1.0) | 36 (0.8) | 15 (0.4) | 0.046 | 28 (0.7) | 36 (0.9) | 0.022 | 18 (0.5) | 15 (0.4) | 0.013 |
| Migraine | 12 (0.1) | 5 (0.1) | 2 (0.1) | 0.013 | 4 (0.1) | 4 (0.1) | <0.001 | 1 (0.0) | 2 (0.1) | 0.014 |
| Mental health disorders | 1260 (6.3) | 154 (3.6) | 148 (4.4) | 0.082 | 154 (3.7) | 154 (3.7) | <0.001 | 146 (4.4) | 148 (4.4) | 0.003 |
| Liver diseases | 189 (0.9) | 22 (0.5) | 13 (0.4) | 0.046 | 17 (0.4) | 22 (0.5) | 0.018 | 13 (0.4) | 13 (0.4) | <0.001 |
| Medications |  |  |  |  |  |  |  |  |  |  |
| Renin-angiotensin-system agents | 4051 (20.2) | 503 (11.8) | 477 (14.2) | 0.154 | 514 (12.3) | 503 (12.0) | 0.008 | 459 (13.7) | 476 (14.2) | 0.015 |
| Beta blockers | 2909 (14.5) | 315 (7.4) | 280 (8.3) | 0.153 | 306 (7.3) | 314 (7.5) | 0.007 | 286 (8.5) | 280 (8.4) | 0.006 |
| Calcium channel blockers | 6261 (31.2) | 810 (19.0) | 787 (23.4) | 0.189 | 795 (19.0) | 810 (19.4) | 0.009 | 773 (23.1) | 786 (23.4) | 0.009 |
| Diuretics | 1657 (8.3) | 126 (3.0) | 116 (3.4) | 0.155 | 113 (2.7) | 125 (3.0) | 0.017 | 110 (3.3) | 116 (3.5) | 0.01 |
| Nitrates | 684 (3.4) | 56 (1.3) | 45 (1.3) | 0.092 | 59 (1.4) | 56 (1.3) | 0.006 | 36 (1.1) | 45 (1.3) | 0.025 |
| Lipid lowering agents | 5547 (27.7) | 750 (17.6) | 752 (22.3) | 0.161 | 756 (18.1) | 749 (17.9) | 0.004 | 763 (22.8) | 752 (22.4) | 0.008 |
| Insulins | 796 (4.0) | 58 (1.4) | 55 (1.6) | 0.109 | 52 (1.2) | 58 (1.4) | 0.013 | 51 (1.5) | 55 (1.6) | 0.01 |
| Antidiabetic drugs | 3380 (16.8) | 426 (10.0) | 429 (12.7) | 0.135 | 431 (10.3) | 426 (10.2) | 0.004 | 446 (13.3) | 428 (12.8) | 0.016 |
| Antiarrhythmic drugs | 86 (0.4) | 8 (0.2) | 9 (0.3) | 0.029 | 10 (0.2) | 8 (0.2) | 0.01 | 9 (0.3) | 9 (0.3) | <0.001 |
| Cardiac glycosides | 126 (0.6) | 8 (0.2) | 9 (0.3) | 0.047 | 12 (0.3) | 8 (0.2) | 0.02 | 11 (0.3) | 9 (0.3) | 0.011 |
| Antiplatelets | 2557 (12.7) | 270 (6.3) | 255 (7.6) | 0.147 | 270 (6.5) | 270 (6.5) | <0.001 | 233 (7.0) | 255 (7.6) | 0.025 |
| Antifibrinolytics and hemostatic | 529 (2.6) | 35 (0.8) | 23 (0.7) | 0.103 | 39 (0.9) | 35 (0.8) | 0.01 | 41 (1.2) | 23 (0.7) | 0.055 |
| Hormonal agents | 8423 (42.0) | 2860 (67.1) | 2280 (67.8) | 0.357 | 2773 (66.4) | 2785 (66.7) | 0.006 | 2258 (67.4) | 2267 (67.6) | 0.006 |
| Glucocorticoids | 3600 (17.9) | 267 (6.3) | 181 (5.4) | 0.267 | 279 (6.7) | 267 (6.4) | 0.012 | 163 (4.9) | 181 (5.4) | 0.024 |
| Antidepressants | 1142 (5.7) | 184 (4.3) | 139 (4.1) | 0.048 | 186 (4.5) | 184 (4.4) | 0.002 | 137 (4.1) | 139 (4.1) | 0.003 |
| Bevacizumab | 516 (2.6) | 28 (0.7) | 19 (0.6) | 0.109 | 29 (0.7) | 28 (0.7) | 0.003 | 23 (0.7) | 19 (0.6) | 0.015 |
| Tranexamic acid | 521 (2.6) | 35 (0.8) | 23 (0.7) | 0.101 | 39 (0.9) | 35 (0.8) | 0.01 | 41 (1.2) | 23 (0.7) | 0.055 |
| Intravenous immunoglobulin | 59 (0.3) | 8 (0.2) | 4 (0.1) | 0.026 | 5 (0.1) | 8 (0.2) | 0.018 | 3 (0.1) | 4 (0.1) | 0.009 |
| NSAIDs | 1506 (7.5) | 347 (8.1) | 260 (7.7) | 0.016 | 341 (8.2) | 341 (8.2) | <0.001 | 246 (7.3) | 257 (7.7) | 0.012 |
| Drugs for gout | 832 (4.1) | 79 (1.9) | 69 (2.1) | 0.09 | 74 (1.8) | 79 (1.9) | 0.009 | 60 (1.8) | 69 (2.1) | 0.02 |
| Antiepileptic drugs | 1105 (5.5) | 131 (3.1) | 90 (2.7) | 0.096 | 140 (3.4) | 130 (3.1) | 0.014 | 93 (2.8) | 90 (2.7) | 0.005 |
| Antiviral drugs | 2745 (13.7) | 357 (8.4) | 275 (8.2) | 0.118 | 344 (8.2) | 356 (8.5) | 0.01 | 279 (8.3) | 275 (8.2) | 0.004 |
| Antibacterial drugs | 4199 (20.9) | 426 (10.0) | 312 (9.3) | 0.22 | 446 (10.7) | 423 (10.1) | 0.018 | 312 (9.3) | 312 (9.3) | <0.001 |
| Immunosuppressants | 1233 (6.1) | 188 (4.4) | 127 (3.8) | 0.073 | 186 (4.5) | 187 (4.5) | 0.001 | 130 (3.9) | 127 (3.8) | 0.005 |
| Adrenaline | 9 (0.0) | 0 (0.0) | 3 (0.1) | 0.03 | 0 (0.0) | 0 (0.0) | <0.001 | 2 (0.1) | 2 (0.1) | <0.001 |
| Anticoagulants | 766 (3.8) | 58 (1.4) | 45 (1.3) | 0.105 | 71 (1.7) | 58 (1.4) | 0.025 | 47 (1.4) | 45 (1.3) | 0.005 |

SMD: Standardized mean difference; SD: Standard deviation; NSAIDs: Non-steroidal anti-inflammatory drugs

**Table S4. Baseline characteristics of patients with a history of cancer before and after propensity score matching**

|  | Before matching | | | | Matched BNT162b2 | | | Matched CoronaVac | | |
| --- | --- | --- | --- | --- | --- | --- | --- | --- | --- | --- |
|  | Unvaccinated | BNT162b2 | CoronaVac | SMD ^a^ | Unvaccinated | BNT162b2 | SMD | Unvaccinated | CoronaVac | SMD |
| n | 29028 | 9171 | 8993 |  | 9006 | 9006 |  | 8929 | 8929 |  |
| Age (mean (SD)), year ^b^ | 67.81 (13.80) | 59.40 (13.39) | 63.31 (11.76) | 0.426 | 59.76 (14.38) | 59.82 (13.03) | 0.004 | 63.21 (14.34) | 63.42 (11.69) | 0.016 |
| Sex = M | 13373 (46.1) | 4084 (44.5) | 4435 (49.3) | 0.064 | 4008 (44.5) | 4018 (44.6) | 0.002 | 4470 (50.1) | 4414 (49.4) | 0.013 |
| Smoking | 432 (1.5) | 79 (0.9) | 109 (1.2) | 0.039 | 76 (0.8) | 79 (0.9) | 0.004 | 102 (1.1) | 109 (1.2) | 0.007 |
| Obesity | 1760 (6.1) | 452 (4.9) | 594 (6.6) | 0.048 | 458 (5.1) | 445 (4.9) | 0.007 | 548 (6.1) | 584 (6.5) | 0.017 |
| Covid-19 | 25 (0.1) | 3 (0.0) | 1 (0.0) | 0.024 | 3 (0.0) | 3 (0.0) | <0.001 | 2 (0.0) | 1 (0.0) | 0.009 |
| White blood cells (mean (SD)), 10^9^/L | 6.91 (4.68) | 6.61 (3.39) | 6.68 (3.33) | 0.049 | 6.87 (5.41) | 6.61 (3.40) | 0.059 | 6.84 (4.11) | 6.67 (3.32) | 0.043 |
| Neutrophils (mean (SD)), x10^9^/L | 4.49 (2.86) | 4.20 (2.31) | 4.25 (2.30) | 0.075 | 4.39 (2.71) | 4.20 (2.31) | 0.074 | 4.43 (2.94) | 4.25 (2.30) | 0.068 |
| Counts of hospitalization since 1 Jan, 2018 (mean (SD)) | 4.77 (6.43) | 3.74 (5.40) | 3.84 (4.87) | 0.119 | 3.82 (4.85) | 3.76 (5.43) | 0.011 | 3.92 (4.23) | 3.85 (4.88) | 0.015 |
| Counts of Accident & Emergency attendance since 1 Jan, 2018 (mean (SD)) | 2.01 (2.66) | 1.12 (1.84) | 1.38 (2.05) | 0.263 | 1.13 (1.53) | 1.13 (1.85) | 0.001 | 1.39 (2.03) | 1.38 (2.05) | 0.004 |
| Carcinoma in situ | 666 (2.3) | 542 (5.9) | 538 (6.0) | 0.124 | 417 (4.6) | 485 (5.4) | 0.035 | 405 (4.5) | 494 (5.5) | 0.046 |
| Metastasis | 627 (2.2) | 91 (1.0) | 111 (1.2) | 0.063 | 80 (0.9) | 91 (1.0) | 0.013 | 94 (1.1) | 111 (1.2) | 0.018 |
| Cancer site |  |  |  |  |  |  |  |  |  |  |
| Bladder | 1055 (3.6) | 247 (2.7) | 292 (3.2) | 0.036 | 213 (2.4) | 245 (2.7) | 0.023 | 307 (3.4) | 292 (3.3) | 0.009 |
| Breast | 3303 (11.4) | 1179 (12.9) | 1001 (11.1) | 0.035 | 1261 (14.0) | 1173 (13.0) | 0.029 | 986 (11.0) | 1001 (11.2) | 0.005 |
| Colorectum | 5089 (17.5) | 1158 (12.6) | 1379 (15.3) | 0.092 | 1138 (12.6) | 1157 (12.8) | 0.006 | 1429 (16.0) | 1379 (15.4) | 0.015 |
| Liver | 1625 (5.6) | 305 (3.3) | 331 (3.7) | 0.074 | 326 (3.6) | 303 (3.4) | 0.014 | 339 (3.8) | 331 (3.7) | 0.005 |
| Lung | 2447 (8.4) | 495 (5.4) | 541 (6.0) | 0.08 | 494 (5.5) | 495 (5.5) | <0.001 | 560 (6.3) | 541 (6.1) | 0.009 |
| Nasopharynx | 1208 (4.2) | 386 (4.2) | 396 (4.4) | 0.008 | 399 (4.4) | 383 (4.3) | 0.009 | 402 (4.5) | 396 (4.4) | 0.003 |
| Prostrate | 1860 (6.4) | 833 (9.1) | 901 (10.0) | 0.088 | 844 (9.4) | 819 (9.1) | 0.01 | 850 (9.5) | 887 (9.9) | 0.014 |
| Skin | 910 (3.1) | 290 (3.2) | 276 (3.1) | 0.004 | 265 (2.9) | 287 (3.2) | 0.014 | 254 (2.8) | 276 (3.1) | 0.015 |
| Stomach | 1065 (3.7) | 299 (3.3) | 347 (3.9) | 0.022 | 309 (3.4) | 298 (3.3) | 0.007 | 365 (4.1) | 347 (3.9) | 0.01 |
| Thyroid | 1162 (4.0) | 683 (7.4) | 539 (6.0) | 0.099 | 639 (7.1) | 654 (7.3) | 0.006 | 558 (6.2) | 535 (6.0) | 0.011 |
| Uterus | 1933 (6.7) | 723 (7.9) | 716 (8.0) | 0.033 | 717 (8.0) | 720 (8.0) | 0.001 | 720 (8.1) | 715 (8.0) | 0.002 |
| Solid tumor | 27208 (93.7) | 8532 (93.0) | 8513 (94.7) | 0.045 | 8372 (93.0) | 8392 (93.2) | 0.009 | 8448 (94.6) | 8450 (94.6) | 0.001 |
| Hematological malignancies | 1820 (6.3) | 639 (7.0) | 480 (5.3) | 0.045 | 634 (7.0) | 614 (6.8) | 0.009 | 481 (5.4) | 479 (5.4) | 0.001 |
| Comorbidities |  |  |  |  |  |  |  |  |  |  |
| Congestive Heart Failure | 629 (2.2) | 38 (0.4) | 56 (0.6) | 0.106 | 41 (0.5) | 38 (0.4) | 0.005 | 57 (0.6) | 56 (0.6) | 0.001 |
| Hypertension | 11354 (39.1) | 2351 (25.6) | 2964 (33.0) | 0.194 | 2362 (26.2) | 2344 (26.0) | 0.005 | 2877 (32.2) | 2949 (33.0) | 0.017 |
| Diabetes | 5745 (19.8) | 1037 (11.3) | 1380 (15.3) | 0.157 | 1009 (11.2) | 1034 (11.5) | 0.009 | 1330 (14.9) | 1375 (15.4) | 0.014 |
| Systemic embolism | 20 (0.1) | 2 (0.0) | 2 (0.0) | 0.015 | 1 (0.0) | 2 (0.0) | 0.009 | 1 (0.0) | 2 (0.0) | 0.009 |
| Vascular disease | 1882 (6.5) | 300 (3.3) | 373 (4.1) | 0.1 | 315 (3.5) | 299 (3.3) | 0.01 | 360 (4.0) | 371 (4.2) | 0.006 |
| Peripheral vascular disease | 215 (0.7) | 21 (0.2) | 26 (0.3) | 0.05 | 18 (0.2) | 21 (0.2) | 0.007 | 26 (0.3) | 26 (0.3) | <0.001 |
| Cerebrovascular disease | 2020 (7.0) | 212 (2.3) | 308 (3.4) | 0.15 | 183 (2.0) | 212 (2.4) | 0.022 | 299 (3.3) | 308 (3.4) | 0.006 |
| Chronic obstructive pulmonary disease | 1198 (4.1) | 189 (2.1) | 195 (2.2) | 0.08 | 189 (2.1) | 187 (2.1) | 0.002 | 181 (2.0) | 195 (2.2) | 0.011 |
| Dementia | 148 (0.5) | 2 (0.0) | 11 (0.1) | 0.067 | 4 (0.0) | 2 (0.0) | 0.012 | 8 (0.1) | 11 (0.1) | 0.01 |
| Paralysis | 69 (0.2) | 4 (0.0) | 3 (0.0) | 0.038 | 3 (0.0) | 4 (0.0) | 0.006 | 2 (0.0) | 3 (0.0) | 0.007 |
| Chronic renal failure | 919 (3.2) | 79 (0.9) | 113 (1.3) | 0.111 | 77 (0.9) | 79 (0.9) | 0.002 | 102 (1.1) | 113 (1.3) | 0.011 |
| Ulcers | 969 (3.3) | 175 (1.9) | 188 (2.1) | 0.06 | 173 (1.9) | 175 (1.9) | 0.002 | 184 (2.1) | 187 (2.1) | 0.002 |
| Rheumatoid arthritis and other inflammatory polyarthropathies | 141 (0.5) | 26 (0.3) | 34 (0.4) | 0.022 | 23 (0.3) | 25 (0.3) | 0.004 | 31 (0.3) | 34 (0.4) | 0.006 |
| Respiratory infections | 2339 (8.1) | 267 (2.9) | 337 (3.7) | 0.153 | 270 (3.0) | 266 (3.0) | 0.003 | 334 (3.7) | 337 (3.8) | 0.002 |
| Viral infections | 245 (0.8) | 41 (0.4) | 46 (0.5) | 0.033 | 38 (0.4) | 40 (0.4) | 0.003 | 40 (0.4) | 46 (0.5) | 0.01 |
| Migraine | 19 (0.1) | 10 (0.1) | 7 (0.1) | 0.01 | 13 (0.1) | 10 (0.1) | 0.009 | 6 (0.1) | 7 (0.1) | 0.004 |
| Mental health disorders | 2158 (7.4) | 432 (4.7) | 455 (5.1) | 0.076 | 411 (4.6) | 429 (4.8) | 0.009 | 435 (4.9) | 454 (5.1) | 0.01 |
| Liver diseases | 476 (1.6) | 77 (0.8) | 77 (0.9) | 0.048 | 79 (0.9) | 77 (0.9) | 0.002 | 78 (0.9) | 77 (0.9) | 0.001 |
| Medications |  |  |  |  |  |  |  |  |  |  |
| Renin-angiotensin-system agents | 6656 (22.9) | 1328 (14.5) | 1574 (17.5) | 0.145 | 1330 (14.8) | 1320 (14.7) | 0.003 | 1572 (17.6) | 1569 (17.6) | 0.001 |
| Beta blockers | 4814 (16.6) | 884 (9.6) | 988 (11.0) | 0.138 | 919 (10.2) | 883 (9.8) | 0.013 | 969 (10.9) | 986 (11.0) | 0.006 |
| Calcium channel blockers | 9885 (34.1) | 2032 (22.2) | 2603 (28.9) | 0.178 | 2003 (22.2) | 2027 (22.5) | 0.006 | 2523 (28.3) | 2591 (29.0) | 0.017 |
| Diuretics | 2162 (7.4) | 236 (2.6) | 298 (3.3) | 0.151 | 247 (2.7) | 236 (2.6) | 0.008 | 286 (3.2) | 298 (3.3) | 0.008 |
| Nitrates | 1322 (4.6) | 169 (1.8) | 214 (2.4) | 0.104 | 165 (1.8) | 169 (1.9) | 0.003 | 198 (2.2) | 213 (2.4) | 0.011 |
| Lipid lowering agents | 9454 (32.6) | 2087 (22.8) | 2566 (28.5) | 0.147 | 2106 (23.4) | 2075 (23.0) | 0.008 | 2556 (28.6) | 2548 (28.5) | 0.002 |
| Insulins | 1112 (3.8) | 118 (1.3) | 169 (1.9) | 0.109 | 118 (1.3) | 118 (1.3) | <0.001 | 177 (2.0) | 169 (1.9) | 0.007 |
| Antidiabetic drugs | 5258 (18.1) | 999 (10.9) | 1323 (14.7) | 0.138 | 984 (10.9) | 996 (11.1) | 0.004 | 1299 (14.5) | 1317 (14.7) | 0.006 |
| Antiarrhythmic drugs | 106 (0.4) | 12 (0.1) | 20 (0.2) | 0.032 | 14 (0.2) | 12 (0.1) | 0.006 | 17 (0.2) | 20 (0.2) | 0.007 |
| Cardiac glycosides | 248 (0.9) | 26 (0.3) | 40 (0.4) | 0.051 | 21 (0.2) | 26 (0.3) | 0.011 | 40 (0.4) | 40 (0.4) | <0.001 |
| Antiplatelets | 4487 (15.5) | 706 (7.7) | 870 (9.7) | 0.163 | 693 (7.7) | 704 (7.8) | 0.005 | 843 (9.4) | 869 (9.7) | 0.01 |
| Antifibrinolytics and hemostatic | 430 (1.5) | 38 (0.4) | 52 (0.6) | 0.074 | 39 (0.4) | 38 (0.4) | 0.002 | 59 (0.7) | 52 (0.6) | 0.01 |
| Hormonal agents | 227 (0.8) | 90 (1.0) | 55 (0.6) | 0.028 | 73 (0.8) | 83 (0.9) | 0.012 | 50 (0.6) | 55 (0.6) | 0.007 |
| Glucocorticoids | 783 (2.7) | 88 (1.0) | 107 (1.2) | 0.087 | 94 (1.0) | 88 (1.0) | 0.007 | 100 (1.1) | 107 (1.2) | 0.007 |
| Antidepressants | 1615 (5.6) | 394 (4.3) | 371 (4.1) | 0.045 | 373 (4.1) | 391 (4.3) | 0.01 | 372 (4.2) | 369 (4.1) | 0.002 |
| Bevacizumab | 0 (0.0) | 0 (0.0) | 0 (0.0) | <0.001 | 0 (0.0) | 0 (0.0) | <0.001 | 0 (0.0) | 0 (0.0) | <0.001 |
| Tranexamic acid | 429 (1.5) | 38 (0.4) | 51 (0.6) | 0.074 | 39 (0.4) | 38 (0.4) | 0.002 | 58 (0.6) | 51 (0.6) | 0.01 |
| Intravenous immunoglobulin | 19 (0.1) | 8 (0.1) | 9 (0.1) | 0.008 | 8 (0.1) | 8 (0.1) | <0.001 | 10 (0.1) | 9 (0.1) | 0.003 |
| NSAIDs ^c^ | 1564 (5.4) | 654 (7.1) | 647 (7.2) | 0.05 | 639 (7.1) | 632 (7.0) | 0.003 | 619 (6.9) | 633 (7.1) | 0.006 |
| Drugs for gout | 1118 (3.9) | 224 (2.4) | 230 (2.6) | 0.054 | 224 (2.5) | 222 (2.5) | 0.001 | 238 (2.7) | 230 (2.6) | 0.006 |
| Antiepileptic drugs | 1054 (3.6) | 218 (2.4) | 158 (1.8) | 0.078 | 218 (2.4) | 218 (2.4) | <0.001 | 132 (1.5) | 158 (1.8) | 0.023 |
| Antiviral drugs | 2141 (7.4) | 510 (5.6) | 560 (6.2) | 0.049 | 511 (5.7) | 509 (5.7) | 0.001 | 570 (6.4) | 560 (6.3) | 0.005 |
| Antibacterial drugs | 2921 (10.1) | 571 (6.2) | 654 (7.3) | 0.094 | 555 (6.2) | 564 (6.3) | 0.004 | 677 (7.6) | 650 (7.3) | 0.012 |
| Immunosuppressants | 0 (0.0) | 0 (0.0) | 0 (0.0) | <0.001 | 0 (0.0) | 0 (0.0) | <0.001 | 0 (0.0) | 0 (0.0) | <0.001 |
| Adrenaline | 11 (0.0) | 0 (0.0) | 5 (0.1) | 0.023 | 0 (0.0) | 0 (0.0) | <0.001 | 4 (0.0) | 5 (0.1) | 0.005 |
| Anticoagulants | 928 (3.2) | 102 (1.1) | 133 (1.5) | 0.097 | 104 (1.2) | 102 (1.1) | 0.002 | 127 (1.4) | 133 (1.5) | 0.006 |

Abbreviations: ^a^ SMD, Standardized mean difference; ^b^ SD, Standard deviation; ^c^ NSAIDs: Non-steroidal anti-inflammatory drugs

**Table S5. Number of 28-days post-vaccination deaths, overall and specific AESI after matching**

|  | Active cancer | | | | History of cancer | | | |
| --- | --- | --- | --- | --- | --- | --- | --- | --- |
|  | Matched BNT162b2 | | Matched CoronaVac | | Matched BNT162b2 | | Matched CoronaVac | |
|  | Unvaccinated | Vaccinated | Unvaccinated | Vaccinated | Unvaccinated | Vaccinated | Unvaccinated | Vaccinated |
|  | 4175 | 4175 | 3352 | 3352 | 9006 | 9006 | 8929 | 8929 |
| Death | 22 (0.5) | 2 (0.0) | 12 (0.4) | 0 (0.0) | 13 (0.1) | 1 (0.0) | 17 (0.2) | 2 (0.0) |
| All AESI | 10 (0.2) | 3 (0.1) | 7 (0.2) | 1 (0.0) | 19 (0.2) | 12 (0.1) | 20 (0.2) | 9 (0.1) |
| Acute aseptic arthritis | 0 (0) | 0 (0) | 0 (0) | 0 (0) | 1 (0.0) | 0 (0.0) | 0 (0.0) | 2 (0.0) |
| Acute disseminated encephalomyelitis (ADEM) | 0 (0) | 0 (0) | 0 (0) | 0 (0) | 0 (0) | 0 (0) | 0 (0) | 0 (0) |
| Anaphylaxis | 0 (0) | 0 (0) | 0 (0) | 0 (0) | 0 (0.0) | 1 (0.0) | 0 (0.0) | 1 (0.0) |
| Anosmia, ageusia | 0 (0) | 0 (0) | 0 (0) | 0 (0) | 0 (0) | 0 (0) | 0 (0) | 0 (0) |
| Acute respiratory distress syndrome | 1 (0.0) | 1 (0.0) | 1 (0.0) | 0 (0.0) | 4 (0.0) | 1 (0.0) | 3 (0.0) | 1 (0.0) |
| Arrhythmia | 2 (0.0) | 2 (0.0) | 1 (0.0) | 0 (0.0) | 1 (0.0) | 4 (0.0) | 2 (0.0) | 2 (0.0) |
| Bell’s Palsy | 0 (0) | 0 (0) | 0 (0) | 0 (0) | 0 (0) | 0 (0) | 0 (0) | 0 (0) |
| Coronary artery disease | 0 (0) | 0 (0) | 2 (0.1) | 0 (0.0) | 0 (0.0) | 2 (0.0) | 1 (0.0) | 0 (0.0) |
| Chilblain - like lesions | 0 (0) | 0 (0) | 0 (0) | 0 (0) | 0 (0) | 0 (0) | 0 (0) | 0 (0) |
| Generalized convulsion | 1 (0.0) | 0 (0.0) | 0 (0) | 0 (0) | 1 (0.0) | 0 (0.0) | 1 (0.0) | 0 (0.0) |
| Erythema multiforme | 0 (0) | 0 (0) | 0 (0) | 0 (0) | 0 (0) | 0 (0) | 0 (0) | 0 (0) |
| Guillain-Barré Syndrome | 0 (0) | 0 (0) | 0 (0) | 0 (0) | 0 (0) | 0 (0) | 0 (0) | 0 (0) |
| Hemorrhagic disease | 0 (0) | 0 (0) | 0 (0) | 0 (0) | 3 (0.0) | 0 (0.0) | 0 (0) | 0 (0) |
| Heart failure | 1 (0.0) | 0 (0.0) | 2 (0.1) | 0 (0.0) | 0 (0) | 0 (0) | 1 (0.0) | 0 (0.0) |
| Acute kidney injury | 1 (0.0) | 0 (0.0) | 0 (0) | 0 (0) | 0 (0.0) | 1 (0.0) | 1 (0.0) | 0 (0.0) |
| Acute liver injury | 0 (0) | 0 (0) | 0 (0) | 0 (0) | 0 (0) | 0 (0) | 0 (0) | 0 (0) |
| Meningoencephalitis | 0 (0) | 0 (0) | 0 (0) | 0 (0) | 0 (0) | 0 (0) | 0 (0) | 0 (0) |
| Myocardial infarction | 0 (0) | 0 (0) | 2 (0.1) | 0 (0.0) | 4 (0.0) | 2 (0.0) | 3 (0.0) | 3 (0.0) |
| Microangiopathy | 0 (0) | 0 (0) | 0 (0) | 0 (0) | 0 (0) | 0 (0) | 0 (0) | 0 (0) |
| Myocarditis | 0 (0) | 0 (0) | 0 (0) | 0 (0) | 1 (0.0) | 0 (0.0) | 0 (0) | 0 (0) |
| Narcolepsy | 0 (0) | 0 (0) | 0 (0) | 0 (0) | 2 (0.0) | 1 (0.0) | 1 (0.0) | 0 (0.0) |
| Acute pancreatitis | 0 (0) | 0 (0) | 0 (0) | 0 (0) | 2 (0.0) | 0 (0.0) | 1 (0.0) | 0 (0.0) |
| Rhabdomyolysis | 0 (0) | 0 (0) | 0 (0) | 0 (0) | 0 (0) | 0 (0) | 0 (0) | 0 (0) |
| Single Organ Cutaneous Vasculitis | 0 (0) | 0 (0) | 0 (0) | 0 (0) | 0 (0) | 0 (0) | 0 (0) | 0 (0) |
| Stress cardiomyopathy | 0 (0) | 0 (0) | 0 (0) | 0 (0) | 0 (0) | 0 (0) | 0 (0) | 0 (0) |
| Sudden death | 3 (0.1) | 0 (0.0) | 1 (0.0) | 0 (0.0) | 0 (0) | 0 (0) | 1 (0.0) | 0 (0.0) |
| Type 1 Diabetes | 0 (0) | 0 (0) | 0 (0) | 0 (0) | 0 (0) | 0 (0) | 0 (0) | 0 (0) |
| (Idiopathic) Thrombocytopenia | 0 (0) | 0 (0) | 0 (0) | 0 (0) | 0 (0) | 0 (0) | 0 (0) | 0 (0) |
| Thromboembolism | 2 (0.0) | 0 (0.0) | 2 (0.1) | 1 (0.0) | 4 (0.0) | 3 (0.0) | 10 (0.1) | 3 (0.0) |
| Subacute thyroiditis | 0 (0) | 0 (0) | 0 (0) | 0 (0) | 0 (0) | 0 (0) | 0 (0) | 0 (0) |
| Transverse myelitis | 0 (0) | 0 (0) | 0 (0) | 0 (0) | 0 (0) | 0 (0) | 0 (0) | 0 (0) |

AESI: adverse events of special interest

**Table S6. Number of 28-days post-vaccination AESI cases following the first dose of vaccination**

|  | Total number of patients | AESI cases | Cumulative incidence rate (%) | χ2 (p-value) |
| --- | --- | --- | --- | --- |
| One-dose only | 3909 | 18 | 0.46 | 0.63 (0.43) |
| Unvaccinated | 49089 | 186 | 0.38 |  |

AESI: adverse events of special interest
